# Supplementary material for: Age of first diagnosis and incidence rate of uterine fibroids in Ghana. A retrospective cohort study
Source: PLoS One. 2023 Mar 16;18(3):e0283201. doi: 10.1371/journal.pone.0283201 (PMC10019645; doi:10.1371/journal.pone.0283201)
Supplement: Attachment — Submitted filename: Review comments_21.09.2022.docx [file pone.0283201.s002.docx]

**Age of First Diagnosis and Incidence Rate of Uterine Fibroids in Ghana. A Retrospective Cohort Study.**

**Title**

- Please review the title, the study design and what was done do not match. Is this relay a cohort study?

**English language check**

- Language is not clear some sentences could be paraphrased. Please use clear unambiguous professional English throughout the manuscript.

**Abstract**

- The first sentence should be paraphrased (line 37-39).
- Please stick to using one term throughout the text for consistency and easy flow of ideas (uterine fibroids). Other terms can be introduced and left as such.
- Clearly describe this as a disorder
- Our setting (Line 40)- be specific is it rural or urban?

**Introduction**

- The introduction is not well written.
- Provide some few statistics on what is happening globally, in Africa and in Ghana to give a clear picture of what is currently happening.
- Line 66-67-the sentences is not clear, paraphrase.
- Line 67-68 work on the flow of ideas, this does not read well.
- Line 76-77 be more specific, is it possible to link the sentence to line 64-65.
- Paraphrase line 82-84.
- The aim could be highlighted or overemphasized for clarity.

**Methods**

- Describe the study design fully
- Paraphrase line 106-108, “assessed” is not the correct term to use.
- Line 116-118 restructure the sentence does not read well.
- Line 123-126 paraphrase
- Line 127 “consecutively selected” use a better term
- Justify why this is a cohort study

**Ethics**

- Line 156 please give the official name of the ethical review committee.

**Results**

- For tables that do not fit in one page enable repeat table titles.
- Line 164-165, line 181“the rests” paraphrase
- Results are fairly well presented.

**Discussion**

- Line 213-214 add references
- Line 276-277 not clear
- Reference lines 234,239 and 241.
- Consider restructuring the discussion and paraphrasing the sentences for easy flow of ideas.
- Note that there is comparability of the studies referenced, your study was only covering South Central Ghana and most of the studies were national.

**Limitations**

- Not fully explained.
- What are the strengths of the study?

**Conclusion**

- It is fairly well stated and linked to the aim.

**General comment**

- The writing style and the English language can be improved to ensure that the paper can be clearly understood by the international audience, the current phrasing makes comprehension difficult.
- Citations can be presented better to help with the flow of ideas and to avoid repetition.

Reference

- Consistency in presentation (i.e. highlighting and underlining)
